# Hydrogen sulfide: the gas that fuels longevity

**DOI:** 10.20517/jca.2022.16

**Published:** 2022-04-14

**Authors:** Erik A. Blackwood, Christopher C. Glembotski

**Affiliations:** Department of Internal Medicine, University of Arizona College of Medicine - Phoenix, Phoenix, AZ 85004, USA.

**Keywords:** ATF4, mTORC1, protein synthesis, hydrogen sulfide, longevity

## Abstract

The molecular determinants of lifespan can be examined in animal models with the long-term objective of applying what is learned to the development of strategies to enhance longevity in humans. Here, we comment on a recent publication examining the molecular mechanisms that determine lifespan in worms, *Caenorhabditis elegans* (*C. elegans*), where it was shown that inhibiting protein synthesis increased levels of the transcription factor, ATF4. Gene expression analyses showed that ATF4 increased the expression of genes responsible for the formation of the gas, hydrogen sulfide (H_2_S). Further examination showed that H_2_S increased longevity in *C. elegans* by modifying proteins in ways that stabilize their structures and enhance their functions. H_2_S has been shown to improve cardiovascular performance in mouse models of heart disease, and clinical trials are underway to test the effects of H_2_S on cardiovascular health in humans. These findings support the concept that nutrient deprivation, which slows protein synthesis and leads to ATF4-mediated H_2_S production, may extend lifespan by improving the function of the cardiovascular system and other systems that influence longevity in humans.

A major independent risk factor for the development of cardiovascular disease is age, which is especially important as the average age of people continues to increase in many parts of the world. To develop therapeutics for the increasing numbers of elderly patients with heart disease, it is important to understand the molecular mechanisms of aging in the cardiovascular system. Like most biological processes, aging can be studied at the cell and molecular levels. Many such studies use experimental models, such as *Drosophila melanogaster* (fruit flies), *C. elegans* (worms) and mice. These studies have shown that age increases defects in genetic and protein networks, leading to a functional and structural decline of the myocardium and vasculature, due partly to increased sensitivity of the cardiovascular system to oxidative stress and inflammation^[1]^. Increased anabolic energy metabolism is associated with decreased longevity, indicating that decreasing anabolic metabolism, thus increasing the catabolic breakdown of stored nutrients, should extend lifespan^[2]^. In this regard, the inhibition of mTOR (mechanistic target of rapamycin), a kinase that plays a central role as a master regulator of anabolic energy metabolism, has been shown to increase lifespan in *C. elegans*, as well as *Drosophila* and mice [Figure 1A].

mTOR is composed of two distinct complexes, mTOR complex 1 and 2 (mTORC1 and mTORC2)^[3]^. mTORC1 regulates cellular growth processes in response to nutrient availability, while mTORC2 regulates cell proliferation and survival; moreover, mTORC1 is a central regulator of metabolic function in essentially all cell and tissue types across many species studied to date^[3]^. One aspect of growth dependent upon mTORC1 activation is the translation of mRNA into protein^[3]^. When nutrients are abundant, mTORC1 activation leads to an anabolic increase of the molecular building blocks of growth. However, when nutrients are limited, such as during dietary restriction, mTORC1 activity decreases, leading to a catabolic breakdown of many biomolecules [Figure 1B]. Genetic and pharmacological evidence shows that it is as a regulator of protein synthesis that mTORC1 acts as a major driver of aging, such that decreasing mTORC1 activity and protein synthesis increases lifespan^[3]^. This makes metabolic sense, since decreased protein synthesis allows the protein-folding machinery to focus on fewer proteins, thus enhancing proteome quality, cellular function and viability.

Nutrient deprivation decreases protein synthesis by inducing the integrated stress response (ISR), a central regulator of protein homeostasis. The main feature of the ISR is a series of kinases, including general control non-derepresible 2 (GCN2), that phosphorylate the protein translation elongation factor, eIF2a [Figure 1C]; phosphorylated eIF2a does not participate in, and actually inhibits most mRNA translation^[3]^. Paradoxically, eIF2a-P increases the translation of a small group of mRNAs that are not translated during unstressed times, but are instead translated only during stresses, such as nutrient deprivation. One such mRNA encodes the transcription factor, ATF4, an important inducer of genes that re-establish protein homeostasis during stress [Figure 1D]. In this way, the decrease in translation of most mRNAs alleviates the protein synthesis and folding load during times of stress, while permitting the translation of a small group of mRNAs that encode proteins, like ATF4 [Figure 1E], that are required to improve stress responses and to restore protein quality, thus avoiding potentially lethal proteotoxicity. While ATF4 has a well-recognized function in decreasing proteotoxic stress, the role of ATF4 in extending lifespan is unknown.

In a recent paper, Statzer *et al.*^[4]^ showed that lifespan in *C. elegans* was extended by decreasing mRNA translation, either by inhibiting mTOR or inhibiting translation directly [Figure 1F]; these effects were found to be ATF4-dependent. While it is well established that nutrient deprivation activates the ISR and subsequent phosphorylation of eIF2α, a necessary step in canonical ATF4 translation [Figure 1D], unexpectedly, this same study demonstrated that ATF4 translation, as well as lifespan extension, occurred in *C. elegans* independently of eIF2α phosphorylation, suggesting a novel non-canonical mechanism for increasing ATF4 translation [Figure 1G]. While this was an intriguing finding, the authors did not pursue it; instead, they focused on exploring the mechanism by which ATF4 affects lifespan; in this regard, they identified a number of ATF4-inducible genes, including those that increase the conversion of cysteine and methionine to hydrogen sulfide (H_2_S), e.g., cystathionine-γ lyase-2 (CTH-2; CGL) [Figure 1H]. They showed that CTH-2 and H_2_S, were required for the lifespan extension in response to inhibition of translation^[4]^ [Figure 1I]. Interestingly, in a publication preceding this study, it was shown that ATF4 activation in response to dietary restriction in mice induced CTH-2 induction and increased H_2_S, which promoted angiogenesis^[5]^. While the authors did not comment on the longevity of the animals as a function of ATF4 in that previous study, its activation was shown to be dependent upon the ISR and phosphorylation of eIF2α by GCN2. This finding appears to differ from that in the present study using *C. elegans*, leading to questions about whether the eIF2a phosphorylation-independence of ATF4 translation in *C. elegans* is specific to that species.

To further examine roles for each mTOR complex, Statzer *et al.*^[4]^ showed that direct inhibition of either mTORC1 or mTORC2 promoted lifespan extension by increasing H_2_S. Interestingly, they found that ATF4 was dispensible for lifespan extension when *C. elegans* were exposed to a liquid culture dietary restriction protocol, or treated with rapamycin, which can inhibit both mTORC1 and mTORC2. Also of note, direct mTORC2 inhibition exhibited a more profound effect on both lifespan extension and H_2_S production, and while the authors focused on mTORC1 signaling, they indicated that further investigation in *C. elegans* and mammalian model systems were needed to decipher the specific roles of mTORC1 and mTORC2 in H_2_S production.

H_2_S is a potent endogenous gasotransmitter that converts protein cysteine thiols and protein sulfenic acids to persulfides in a process called persulfidation [Figure 1J] that can adaptively stabilize proteins during stresses that might otherwise compromise protein structure and function^[6]^. Over the last two decades, H_2_S has been shown to be a critical signaling molecule in the cardiovascular system due to its functions in vasodilation and its roles as an antioxidant and in the promotion of protein modifications such as persulfidation^[6]^. H_2_S declines as a function of age, which increases inflammation and a pathological accumulation of reactive oxygen species, as well as impairing adaptive signaling pathways (e.g., AMPK, SIRT1, and NRF2)^[7]^. In the heart, H_2_S activates NRF2 and AMPK, confers cardioprotection via PGC1α, and inhibits mTORC1^[6]^. H_2_S donor molecules (e.g., SG-1002, GYY4137) confer protection against cardiomyocyte death and preserve cardiac function in models of ischemia-reperfusion injury, pressure overload-induced heart failure, and cardiac dysfunction that is secondary to obesity and diabetic metabolic syndromes^[6]^. While the cardioprotective effects of H_2_S are clear, there are some findings that complicate the H_2_S story in the heart. For example, protein persulfidation as a function of age was examined in different tissues in mice subjected to dietary restriction^[8]^. While, in that study, dietary restriction promoted protein persulfidation in the liver, kidney, skeletal muscle and brain, only in the heart did protein persulfidation actually decrease in the same mice. Such a cardiac-specific decrease in H_2_S as a function of age could be due to the enhanced thioredoxin system in the heart, which could lead to increases in both depersulfidation and H_2_S breakdown. Such findings highlight the need for further investigation of the role of H_2_S, as well as protein persulfidation in the aging heart.

Decreasing translation, either directly with pharmacological approaches or indirectly with dietary restriction, has been well demonstrated to increase H_2_S levels and extend lifespan^[6]^. Yet, to date, there have been only two clinical trials testing pure H_2_S donors on age-related cardiovascular disorders (NCT02899364 and NCT02278276); the results of these trials have not yet been published. As efforts continue to enhance the pharmacological properties of H_2_S donors to limit the untoward generation of byproducts during metabolism or increase their pharmacokinetic profile to allow for slow release in chronic disease states and aging, it is important to continue to deepen the mechanistic understanding of H_2_S generation.

Statzer *et al.*^[4]^ have identified a novel mechanism whereby ATF4 increases H_2_S and extends lifespan. These exciting findings could open possibilities for future therapeutic interventions. However, as might be expected with any stress-responsive system, too much ATF4 could be deleterious, as it has been shown that chronic and high levels of ATF4 promote lethal cardiac atrophy via cardiomyocyte apoptosis^[9]^. It might be of interest to explore the feasibility of small molecule inducers of ATF4 translation that act rapidly, yet transiently, which would facilitate better control over ATF4 levels; such an approach has been established by the Wiseman group for other stress-responsive proteins (e.g., ATF6 and IRE1)^[10]^.

In conclusion, while studies such as those reviewed here provide exciting evidence for the possible roles of ATF4 and H_2_S in promoting longevity, like many stress-signaling pathways, their future utility in the aging human population will require extensive studies in order to determine whether there are optimal levels of each that can provide the desired effects on lifespan without risking untoward side effects.

## Figures and Tables

**Figure 1.**
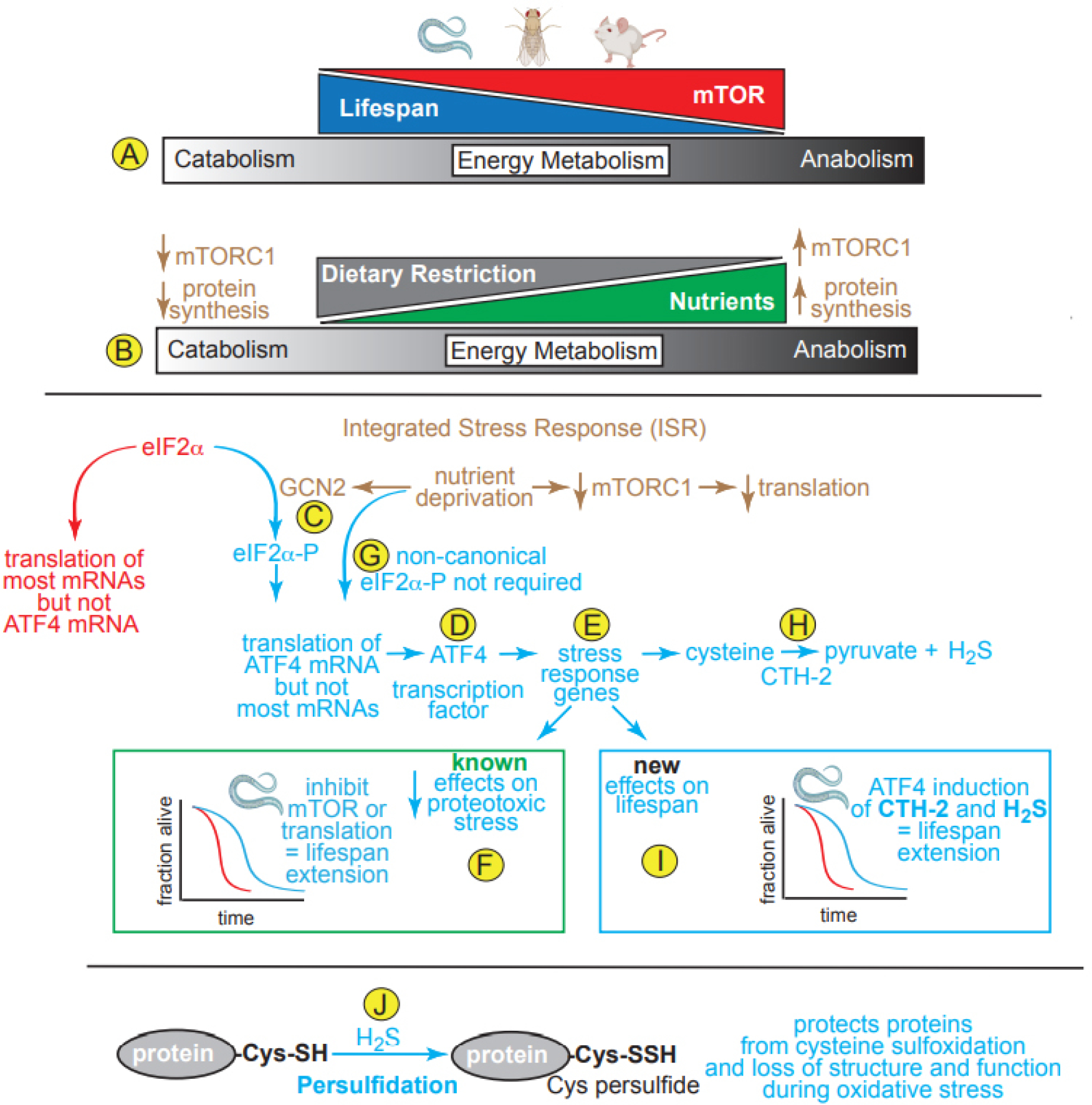
(A, B) Effects on Lifespan of mTOR, protein synthesis and dietary restriction on catabolic and anabolic energy metabolism. (C) The integrated stress response (ISR) involves activation of GCN2, a kinase that is activated upon nutrient deprivation, phosphorylates eIF2a, which inhibits most mRNA translation, but allows translation of a few mRNAs, such as that encoding ATF4. (D) ATF4 is a transcription factor that (E) induces numerous stress-response genes required for cells to adjust to decreased nutrient availability. (F) Inhibition of mTOR, which then decreases translation, or inhibition of translation direction with protein synthesis inhibitors, such as cycloheximide, decreases proteotoxic stress in *C. elegans*, leading to lifespan extension. (G) Statzer *et al.*^[4]^ showed a new, non-canonical mechanism of increasing ATF4 translation that does not require eIF2a phosphorylation. (H) One of the genes Statzer *et al.*^[4]^ found to be induced by ATF4 in *C. elegans* is CTH-2, which catalyzes the formation of H_2_S. (I) Among the new findings reported by Statzer *et al.*^[4]^ are that ATF4 induced by inhibiting mTOR or translation induces CTH-2, which increases H_2_S and lifespan extension; (J) H_2_S contributes to persulfication of cysteine thiols on proteins, which affects their structures and functions in protective ways.

## Data Availability

Not applicable.
